# Current and cumulative malaria infections in a setting embarking on elimination: Amhara, Ethiopia

**DOI:** 10.1186/s12936-017-1884-y

**Published:** 2017-06-08

**Authors:** Woyneshet G. Yalew, Sampa Pal, Pooja Bansil, Rebecca Dabbs, Kevin Tetteh, Caterina Guinovart, Michael Kalnoky, Belendia A. Serda, Berhane H. Tesfay, Belay B. Beyene, Catherine Seneviratne, Megan Littrell, Lindsay Yokobe, Gregory S. Noland, Gonzalo J. Domingo, Asefaw Getachew, Chris Drakeley, Richard W. Steketee

**Affiliations:** 1 Regional Health Research Laboratory Center, Amhara National Regional State Health Bureau, P.O. Box 495, Bahir Dar, Ethiopia; 2 PATH, 2201 Westlake Avenue, Suite 200, Seattle, WA 98121 USA; 3 Faculty of Infectious and Tropical Diseases, London School of Tropical Medicine and Hygiene, Keppel Street, London, WCIE 7HT UK; 4 The Carter Center, 453 Freedom Parkway, Atlanta, GA 30307 USA

**Keywords:** Malaria, *Plasmodium falciparum*, *Plasmodium vivax*, Seroconversion, Seroprevalence, Malaria transmission

## Abstract

**Background:**

Since 2005, Ethiopia has aggressively scaled up malaria prevention and case management. As a result, the number of malaria cases and deaths has significantly declined. In order to track progress towards the elimination of malaria in Amhara Region, coverage of malaria control tools and current malaria transmission need to be documented.

**Methods:**

A cross-sectional household survey oversampling children under 5 years of age was conducted during the dry season in 2013. A bivalent rapid diagnostic test (RDT) detecting both *Plasmodium falciparum* and *Plasmodium vivax* and serology assays using merozoite antigens from both these species were used to assess the prevalence of malaria infections and exposure to malaria parasites in 16 *woredas* (districts) in Amhara Region.

**Results:**

7878 participants were included, with a mean age of 16.8 years (range 0.5–102.8 years) and 42.0% being children under 5 years of age. The age-adjusted RDT-positivity for *P. falciparum* and *P. vivax* infection was 1.5 and 0.4%, respectively, of which 0.05% presented as co-infections. Overall age-adjusted seroprevalence was 30.0% for *P. falciparum*, 21.8% for *P. vivax*, and seroprevalence for any malaria species was 39.4%. The prevalence of RDT-positive infections varied by *woreda*, ranging from 0.0 to 8.3% and by altitude with rates of 3.2, 0.7, and 0.4% at under 2000, 2000–2500, and >2500 m, respectively. Serological analysis showed heterogeneity in transmission intensity by area and altitude and evidence for a change in the force of infection in the mid-2000s.

**Conclusions:**

Current and historic malaria transmission across Amhara Region show substantial variation by age and altitude with some settings showing very low or near-zero transmission. *Plasmodium vivax* infections appear to be lower but relatively more stable across geography and altitude, while *P. falciparum* is the dominant infection in the higher transmission, low-altitude areas. Age-dependent seroprevalence analyses indicates a drop in transmission occurred in the mid-2000s, coinciding with malaria control scale-up efforts. As malaria parasitaemia rates get very low with elimination efforts, serological evaluation may help track progress to elimination.

**Electronic supplementary material:**

The online version of this article (doi:10.1186/s12936-017-1884-y) contains supplementary material, which is available to authorized users.

## Background

Malaria is a major public health challenge in Ethiopia, accounting for approximately 12 million cases each year [1]. Malaria epidemiology in Ethiopia is relatively unique in Africa in that both *Plasmodium falciparum* and *Plasmodium vivax* are present and malaria transmission is generally unstable, with focal, seasonal outbreaks and occasional epidemics [1].

During the last decade, significant scale-up of interventions—including population-wide distribution of free, long-lasting insecticide-treated nets (LLINs), indoor residual spraying (IRS), nationwide roll-out of rapid diagnostic tests (RDTs) and artemisinin-based combination treatments—to reduce malaria transmission was implemented throughout the country [2]. This resulted in a significant reduction in the prevalence of the malarial infection and illness and its consequences [3–6]. It is estimated that, if these activities are continued and sustained in the coming 5 years, 50 million malaria cases will be averted and $39 million in costs associated with patient care and treatment will be saved [7]. It is in this context that there is a renewed commitment to advance toward malaria elimination in Ethiopia.

To facilitate malaria elimination in geographical areas with historically low transmission [8], it is imperative to first document the current levels of malaria transmission and to provide the necessary evidence to extend effective malaria elimination activities. Measuring malaria transmission in low transmission areas is challenging, as microscopy and/or RDT results both underestimate infections and typically present a narrow dynamic range of malaria prevalence estimates, which require large sample sizes [9–11]. While molecular diagnostic tests such as PCR are more sensitive and may capture a more accurate representation of infection prevalence, they are currently impractical in terms of complexity and cost, and in the case of *P. vivax*, require a larger specimen volume [12, 13]. Serological markers measuring cumulative exposure can be a more practical and cost effective alternative, and may provide improved discrimination of transmission rates in the low to medium transmission areas by comparing seroprevalence and seroconversion rates. A recent study based on school surveys from the Oromia Regional State of Ethiopia, demonstrate the value of serological marker-based indicators in low transmission areas (seroprevalence of 0–12.7% for *P. falciparum* and 0–4.5% for *P. vivax*) [14].

A collaborative effort between the Federal Ministry of Health and Amhara National Regional State Health Bureau (ANRSHB); the Malaria Control and Elimination Partnership in Africa (MACEPA), a programme at PATH; and The Carter Center (TCC) was established to develop, implement, and evaluate a comprehensive malaria elimination strategy in selected areas of Amhara Region, Ethiopia. Before the implementation of activities, this study was conducted to estimate 2013 baseline rates of: (1) core malaria interventions including LLIN ownership and use, and IRS coverage; and (2) current and cumulative malaria infections due to *P. falciparum* and/or *P. vivax* using a population-based survey with malaria RDTs and malaria serology.

## Methods

### Study area

**Fig. 1:**
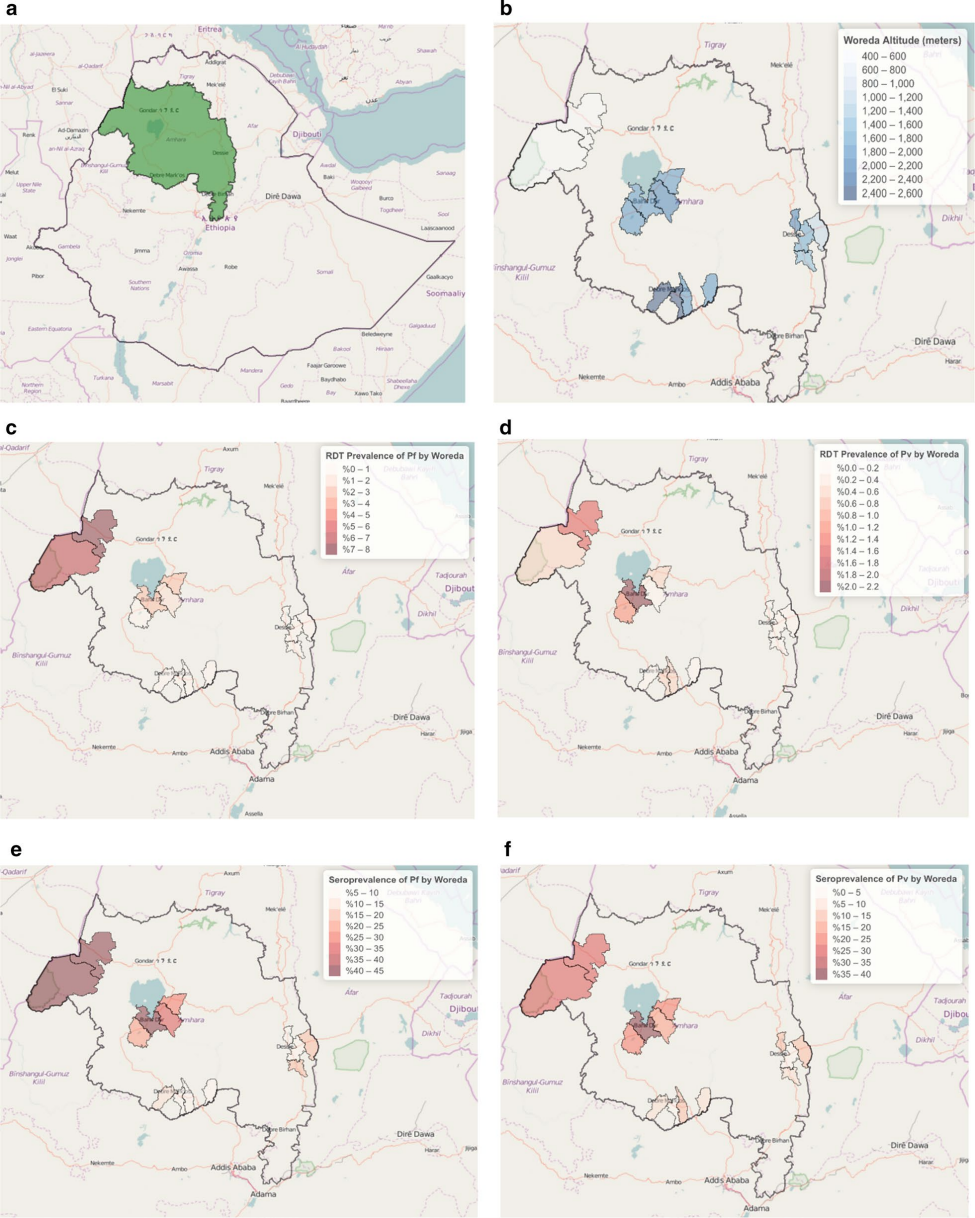
**a** Amhara Region, Ethiopia, **b** survey areas (*woredas)* selected for the Malaria Elimination, colour-coded light to dark from lowest to highest for altitude, **c** *Plasmodium falciparum* RDT prevalence, **d** *Plasmodium vivax* RDT prevalence, **e** *Plasmodium falciparum* seroprevalence, and **f** *Plasmodium vivax* seroprevalence

This study was conducted in Ethiopia’s Amhara Region, which has a population of approximately 20 million people. In 2012, the reported annual incidence of malaria in the Amhara Region was 60 cases per 1000 population, accounting for 19% of the national malaria burden [15, 16]. The Amhara Region has significant variations in altitude, temperature, and annual rainfall resulting in areas of differing malaria risk and transmission for both *P. falciparum* and *P. vivax*. Malaria transmission is seasonal, with the major malaria transmission season being between September and December. For the purpose of this study, *woredas* (districts) of Amhara were divided into four eco-epidemiological zones (very low, very low to low, low, and low to moderate) based on weekly malaria incidence data from 2012. A total of 16 *woredas* from these four unique eco-epidemiological zones were selected (Fig. 1a, b).

### Ethical considerations

This research protocol was reviewed and approved by ethical review committees at Amhara National Regional State (Amhara Research Ethics Review Committee), PATH, and Emory University.

### Study design and data collection

A cross-sectional household survey was conducted in March–April 2013 during the dry, low-transmission season to provide estimates of malaria burden and coverage of malaria control tools at *woreda* level in 16 target *woredas*. *Woreda*-stratified sampling was used to draw a sample from each *woreda* that was proportional to the *woreda* population. Within each *woreda*, enumeration areas (EAs) were selected with probability proportional to size. A total of 278 EAs were selected from *woreda* listings of all EAs developed by the Central Statistical Agency for the 2007 census. In each selected EA, 25 households were selected using simple random sampling from a sampling frame of all households created by EA mapping. EAs were mapped using personal digital assistants (PDAs). Within each sampled household, all children aged 6 months to 4 years old were eligible for malaria testing. Household members over 5 years of age were eligible in one out of every four households. Individuals were invited to participate and were included after verbal informed consent was obtained.

A standardized questionnaire to collect information on socio-demographic characteristics, knowledge and attitudes about malaria, and use of malaria control tools was administered to each participant. Data were collected using PDAs. Finger-prick blood samples were collected from consenting participants to perform an RDT (bivalent SD Bioline Malaria Ag P.f./P.v. HRP-2/pLDH POCT) and prepare dried blood spots (DBS) on filter paper. Participants with a positive RDT result received antimalarial treatment per national guidelines. Those seriously ill were referred to seek immediate care at the nearest health facility.

### Laboratory methods

Samples were assayed for anti-malarial IgG antibody responses to *P. falciparum and P. vivax* merozoite surface protein (MSP-1_19_) and apical membrane antigen 1 (AMA-1) recombinant protein [11] by enzyme-linked immunosorbent assay (ELISA) at the Amhara Region Health Research Laboratory Centre. Three-millimetre diameter circles were punched from DBS and reconstituted in PBS buffer as previously described [10, 17]. Plates were coated with antigens at a concentration of 0.5 μg/mL overnight. Eluates from test samples were added at a dilution of 1:1000 and species-specific positive controls sera were added at serial dilutions on each plate to generate standard curves. After overnight incubation, horseradish-peroxidase-conjugated rabbit-anti-human IgG (DAKO, USA) secondary antibody was added followed by substrate Tetramethylbenzidine (TMB) (SurModics, USA) as color detection. Optical density (OD) values were read at 450 nm after 15 min incubation with the substrate.

### Data analysis

Data analysis was done using Stata (College Station, USA). Estimates of seroprevalence and prevalence of malaria infection by RDT were weighted to account for the sampling design.

Serology samples with duplicate OD values that varied by more than 50% were excluded from the analysis. Duplicate OD results were averaged, the raw ODs were transformed into titres using the standard curve generated by the positive control on each assay plate. Seropositivity was calculated by fitting a Gaussian distribution to normalized OD values using maximum-likelihood methods assuming two distributions, a narrow seronegative, and broad seropositive [18]. For each antigen, the cutoff value was determined by the mean OD of the seronegative population plus three standard deviations [11]. *Plasmodium falciparum* and *P. vivax* seropositivity was defined as an individual being positive for either or both respective antigens, and was analysed by age, altitude, and *woreda*. Simple reverse catalytic models were fitted to seroprevalence and age data using maximum likelihood methods and seroconversion rates (SCR) were estimated [11]. Evidence for models with two forces of infection were investigated using profile likelihood plots and compared with a model with a single force of infection using log-likelihood tests [10].

Logistic regression was employed to determine the likelihood of being RDT-positive for *P. vivax* and/or *P. falciparum* (odds ratios [OR]; 95% confidence intervals [CI]) as compared to having a negative RDT test result, for sex, age, altitude, fever, malaria history, bed net ownership, bed net use, and IRS spraying in the last 12 months. Univariate and multivariate models including all variables to estimate adjusted ORs were run. Two independent models were run for altitude (<2000 and ≥2000 m) because there was a significant effect modification between altitude and bed net use/ownership and altitude and IRS. Moreover, the national malaria programme uses the 2000 m altitude cut-off for targeted deployment of malaria control tools, hence supporting the a priori plan to run the two models separately. Also, it was not possible to add a third altitude band above 2500 m for the regression as was done for the descriptive analysis because the number of RDT positives was too small. Similar regression models were employed for *P. falciparum* and *P. vivax* seropositivity.

## Results

### Study population

A total of 7878 individuals from 16 *woredas* (Fig. 1a, b) participated in the cross-sectional survey and had questionnaire data, RDT malaria blood testing results, and serology results available for analysis. 52.1% of participants were female, 42.0% were less than 4 years of age, and 55.1% lived below 2000 m (Table 1). At the household level, 59.2% owned a bed net and 31.4% had received IRS in the previous 12 months. Only 21.7% of participants reported having used a bed net the previous night, and 11.6% reported having had a fever in the previous 2 weeks. A total of 151 (1.9%) were RDT-positive (age-adjusted prevalence) (Table 2); 120 (1.5%) were RDT-positive for *P. falciparum*, 35 (0.4%) for *P. vivax,* and 4 (0.05%) for both *P. falciparum* and *P. vivax*. Among RDT-positive individuals, 41.0% were asymptomatic (no reported fever in the last 2 weeks), with no significant differences by age group. The overall seroprevalence was 39.4% (n = 3102), with 30.0% (2367) being *P. falciparum* seropositive and 21.8% (1720) *P. vivax* seropositive.

**Table 1:** Characteristics of survey participants (n = 7878)

	Number	Percent (%)
Sex		
Female	4101	52.1
Male	3777	47.9
Age (years)		
6 months–4	3307	42.0
5–9	924	11.7
10–19	1094	13.9
20–39	1486	18.9
40–59	696	8.8
60+	371	4.7
Woreda		
Very low transmission		
Aneded	250	3.2
Awabel	391	5.0
Gozamin	393	5.0
Shebel Berenta	315	4.0
Very low to low transmission		
Bati	396	5.0
Dawa Chef	589	7.5
Kalu	631	8.0
Tehuledere	275	3.5
Low transmission		
Bahir Dar Zuriya	771	9.8
Dera	830	10.5
Fogera	900	11.4
Mecha	1249	15.9
Low to moderate transmission		
Genda Wuha Town	53	0.7
Gulegu	78	1.0
Metema	424	5.4
Quara	333	4.2
Altitude (m)		
<2000	4388	55.1
2000–2500	3175	40.3
>2500	365	4.6
Bed net use the previous night		
No	6165	78.3
Yes	1713	21.7
Fever in last 2 weeks		
No	6951	88.4
Yes	909	11.6

Unweighted estimates

**Table 2:** Prevalence of malaria infection by RDT-positivity and seroprevalence (n = 7878)

	Number	Percent (%)
RDT-positivity		
*P. falciparum*	120	1.5
*P. vivax*	35	0.4
*P. falciparum* and *P. vivax* co-infection	4	0.05
Total *P. falciparum* and/or *P. vivax*	151	1.9
Seroprevalence		
*P. falciparum*	2367	30.0
*P. vivax*	1720	21.8
*P. falciparum* and *P. vivax* co-infection	985	12.5
Total *P. falciparum* and/or *P. vivax*	3102	39.4

Weighted estimates based on sampling strategy

### Geospatial distribution of RDT-positivity and seroprevalence

**Fig. 2:**
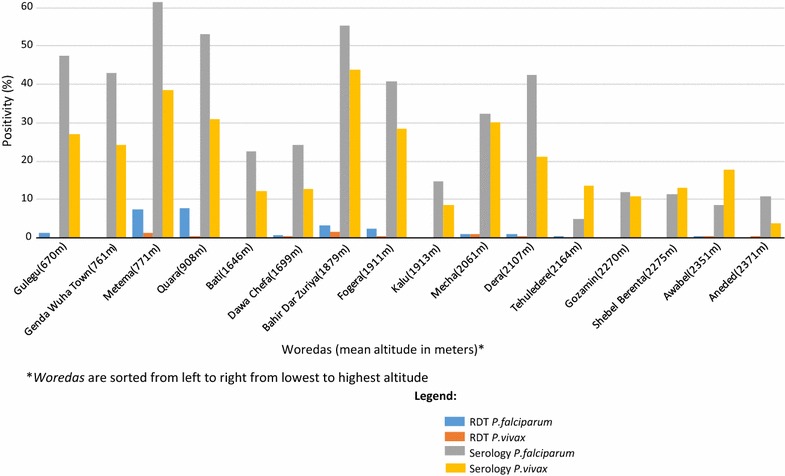
*Plasmodium falciparum* and *P. vivax* RDT-positivity and seroprevalence by *woreda*. The *woredas* are arranged from highest to lowest altitude

**Fig. 3:**
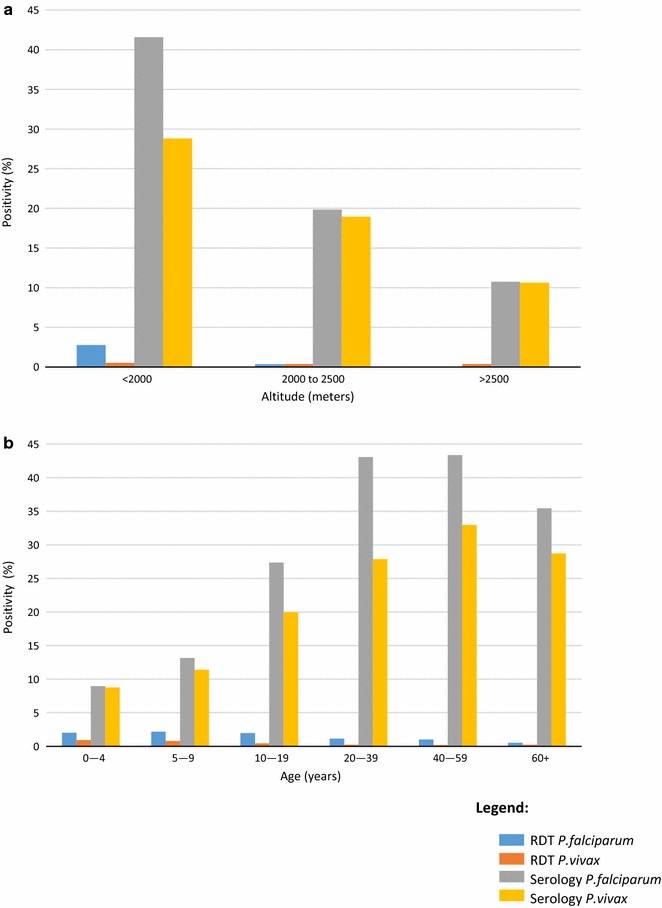
*Plasmodium falciparum* and *P. vivax* RDT-positivity and seroprevalence by **a** altitude and **b** age group

There was substantial variation in RDT-positivity for *P. falciparum* (from <1.0% to roughly 8.0%) (Fig. 1c) and less variation for *P. vivax* (from 0% to roughly 1.5%) (Fig. 1d) over the 16 *woredas*. *Plasmodium falciparum* seroprevalence by *woreda* ranged from 61.5% in Metema to 4.8% in Tehuledere (Fig. 1e); and *P. vivax* seroprevalence by *woreda* ranged from 43.7% in Bahir Dar Zuriya to 3.7% in Aneded (Fig. 1f). Overall, the RDT-positivity rates and the seroprevalence for both *P. falciparum* and *P. vivax* decreased with increasing altitude (Fig. 2). While higher RDT and serology prevalence was observed in *woredas* located at lower altitudes, there was heterogeneity within the different elevation strata (Fig. 2). The RDT-positivity by altitude was 3.2, 0.7, and 0.4% for <2000, 2000–2500, and >2500 m, respectively. Respective RDT-positivity for *P. falciparum* and *P. vivax* at these altitudes were 2.7 and 0.5% (<2000 m), 0.3 and 0.4% (2000–2500 m), and 0 and 0.4% (≥2500 m) (Fig. 3a). Additionally, the respective seroprevalence for *P. falciparum* and *P. vivax* at these altitudes were 41.6 and 28.8% (<2000 m), 19.8 and 18.9% (2000–2500 m), and 10.7 and 10.6% (≥2500 m) (Fig. 3a). There were no significant differences in seroprevalence by gender across the different elevation strata.

### RDT-positivity and seropositivity by age

During this low transmission season, RDT-positivity for both *P. falciparum* and *P. vivax* was highest amongst 0–9-year-olds, after which RDT-positivity decreased with increasing age (Fig. 3b). Across all age groups, *P. falciparum* RDT-positivity was higher than *P. vivax* RDT-positivity.

**Fig. 4:**
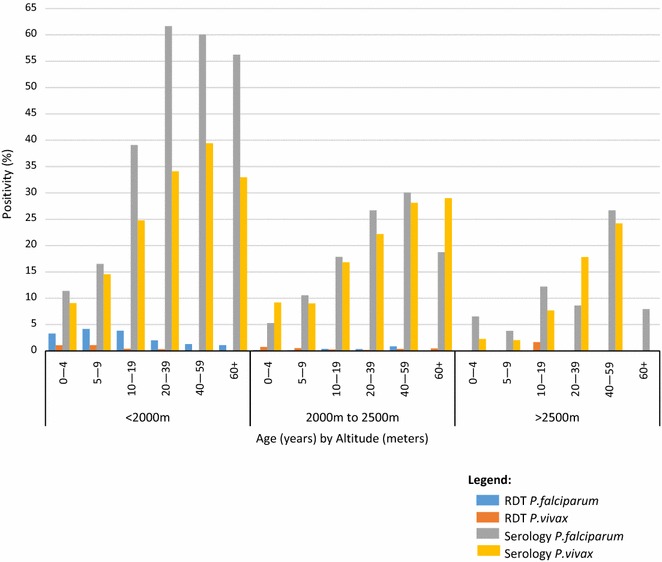
*Plasmodium falciparum* and *P. vivax* RDT-positivity and seroprevalence by altitude and age group

*Plasmodium falciparum* and *P. vivax* seroprevalence increased by age group, although a slight decrease among those aged 60 years and older was observed (Fig. 3b). This general pattern of increasing *P. falciparum* and *P. vivax* seroprevalence by age was seen in each of the altitude strata (Fig. 4). Overall, *P. falciparum* seroprevalence was higher than *P. vivax* seroprevalence across all age groups. However, when stratified by altitude, this pattern was only observed for those living at <2000 m. *Plasmodium falciparum* and *P. vivax* seroprevalences were more similar at higher altitudes, although there was variability observed in the >2500 m stratum, which is likely due to small number of positives per age group.

### Seroconversion rates

**Fig. 5:**
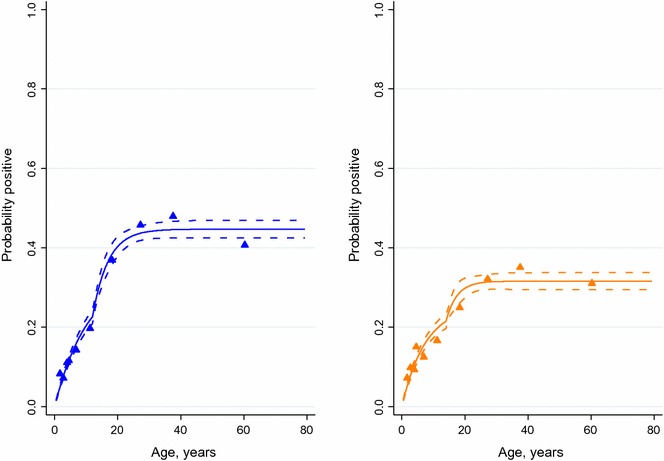
Age-seroconversion plots for antibody responses to *Plasmodium falciparum* antigens (*left*) and *P. vivax* antigens (*right*). *Triangles* represent deciles of observed data, *solid lines* represent the fit to the data of a reverse catalytic conversion model and *broken blue lines* provide the 95% confidence interval for this fit

The age seroconversion plots for antibody responses for *P. falciparum* and *P. vivax* are shown in Fig. 5. The model with two forces of infection (a higher force of infection prior to approximately 15 years ago and a lower force of infection in the last 15 years) provided a better fit to the data than a model with a single force of infection. These models suggest that a substantial reduction in the exposure to both *P. falciparum* and *P. vivax* infections has occurred within the last 10–15 years (*P. falciparum*: current SCR 0.032 year^−1^ [0.029–0.035], previous SCR 0.26 year^−1^ [0.11–0.57]; *P. vivax*: current SCR 0.034 year^−1^ [0.03–0.038], previous SCR 0.22 year^−1^ [0.053–0.91]). The age seroconversion plots for antibody responses for *P. falciparum* and *P. vivax* by altitude strata are shown in Additional file 1, showing similar trends in all altitudes.

The prevalence of RDT-positivity, reported fever and seroprevalence decreased with increasing altitude (Additional file 2). Malaria risk factors associated with RDT-positivity and seroprevalence by altitude (<2000 and ≥2000 m) are shown in Additional files 3 and 4. In both models, individuals with a reported fever in the past 2 weeks were significantly more likely to be RDT-positive, and there was a decreasing risk of RDT-positivity with increasing age. In altitudes below 2000 m, household ownership of a bed net was significantly protective against RDT-positivity; in altitudes ≥2000 m, those with a recent history of malaria were significantly more likely to be RDT-positive, and households that received IRS in the last 12 months were significantly protected against RDT-positivity. For seroprevalence, in altitudes under 2000 m, individuals with reported fever in the previous 2 weeks were significantly more likely to be seropositive, and household ownership of a bed net was significantly protective of seropositivity. In altitudes ≥2000 m, individuals with a history of malaria and households that had received IRS in the previous 12 months were significantly more likely to be seropositive.

Additional file 5 shows that seropositivity for *P. falciparum* is present in younger children across the different elevation strata, and persists even at the highest altitudes (>2500 m). However, all seropositive cases seen above 2500 m are borderline positive, as they are near the limit of detection of 0.02 OD units.

## Discussion

The overall RDT-positivity in the study area during the dry season (March–April) was 1.9%, when the prevalence of infection is expected to be significantly lower than during the major malaria transmission season, which is between September and December in Amhara region. This low prevalence of parasitaemia is probably an underestimate due to the imperfect RDT sensitivity, however it confirms that ongoing transmission persists in this region at a low level into the dry/low transmission season. Another study conducted in July 2013 at the end of the dry season found a high prevalence (12.0%) of RDT-diagnosed *Plasmodium* infection in nearby low-land farming areas of Amhara [19]. The use of small-scale irrigation schemes employed to ensure food security in the area during the dry season might contribute to maintaining the transmission. A recent study found that malaria transmission was significantly higher in irrigated areas and that was primarily due to the higher number of mosquito breeding sites such as waterlogged agricultural field puddles, leakage pools from irrigation canals and poorly functioning irrigation canals [20]. The relatively low use of malaria prevention tools (e.g., 59.2% household bed net ownership and 22% of individuals sleeping under a bed net the previous night) might represent the specific targeting of LLINs to areas <2000 m and the study sample included many living at higher elevations (areas not targeted for LLINs). However, the relatively low use despite ownership highlights the continuous need to educate and increase the awareness that malaria persists year round, and therefore the use of bed nets and other malaria prevention strategies should be reinforced throughout the year [21].

RDT-positivity was highest among children 0–9 years of age, although this pattern was only seen in the lowest altitude areas. The higher prevalence of infection in this young age group is likely to be driven by lower levels of naturally acquired immunity, with higher parasitaemia densities and potentially longer persistence of parasitaemia at a level that could be detected by RDTs [22]. This shows that, even in low transmission areas like this one in Ethiopia, an age pattern reflecting build-up of immunity is observed [23].

Seroprevalence reflects cumulative exposure to malaria, and, with the antigenic targets used in this study, is expected to fluctuate less between the different transmission seasons. As expected, seroprevalence increased with age for both *P. falciparum* and *P. vivax* antigens, reflecting the cumulative exposure to these infections over time, given that, once acquired, antibody responses to MSP-1_19_ and AMA-1 can persist for several years [24]. Thus, seroprevalence in the youngest age group, which ranged from 2.3 to 11.4%, was associated with altitude, and supports the observation of ongoing transmission showed by the RDT results in the same age range. The slight decline in seroprevalence among individuals aged 60 years and older may be due to waning of antibody titers with age, or perhaps reflect decreased exposure to malaria transmission associated with a reduction of farming activities and/or less travel.

The RDT-positivity and the seroprevalence for both *P. falciparum* and *P. vivax* decreased with increasing altitude, which reflects the well-known phenomenon that transmission is generally higher at lower altitudes. In areas below 2000 m, RDT positivity for *P. falciparum* was higher than for *P. vivax* (2.7 and 0.5%, respectively). However, at altitudes over 2500 m the opposite was found: the RDT prevalence for *P. vivax* was 0.4 and 0% for *P. falciparum*, which may be related to the fact that *P. vivax* can have persistent liver stages and infection relapses and is also more tolerant of lower ambient temperatures [25]. Moreover, a recent meta-analysis suggests that the proportion of submicroscopic *P. vivax* infections is higher than for *P. falciparum* particularly in low transmission intensity areas [26].

The 2011 Malaria Indicator Survey (MIS) found the same altitude pattern, with a 13-fold higher prevalence of malaria infection in areas under 2000 m, as compared to higher altitudes [1]. Of note, in the 2011 MIS, at altitudes above 2000 m, *P. falciparum* was not detected by microscopy and *P. falciparum* positivity by RDT was of 0.3%, showing the variation due to the different sensitivities of the tests used and highlighting the challenges of monitoring transmission in very low transmission areas.

The dominance of *P. falciparum* RDT-positivity and seroprevalence in areas under 2000 m suggests that these areas are driving the transmission in the Amhara Region by exporting infections to the higher altitude areas. At elevations over 2000 m, RDT prevalence was almost zero, and *P. falciparum* and *P. vivax* seroprevalences, which were nearly equivalent, were much lower. All age groups showed seropositivity, although seropositive children in areas above 2500 m had very low antibody titers. This suggests that local transmission in higher altitude areas is very low and that exposure to infection is likely to be acquired elsewhere and imported as a result of the population’s mobility and/or migrant labour workforce [27, 28].

Finally, seroconversion rates showed a statistically significant change in the force of malaria infection, suggesting that malaria transmission intensity was higher and was reduced nearly tenfold (from approximately 25% of individuals seroconverting per year previously to 3% currently) in the early-to-mid-2000s in Amhara. This finding probably reflects the success of the recent aggressive scale-up of malaria control activities in the area [7].

In a low transmission setting such as Amhara, the typical malaria age pattern, where younger individuals, especially children, have the highest risk of infections, is also observed. Although reported fever in the past 2 weeks was a significant predictor of RDT-positivity, 63.1 and 46.4% of RDT-positive individuals in altitudes <2000 and ≥2000 m, respectively, did not report a history of fever. This suggests that naturally acquired immunity is attained early in life [22]. Similar results were observed during a population-wide malaria testing and treatment intervention implemented in six villages in Amhara Region, Ethiopia [29].

Household bed net ownership was significantly protective against both RDT positivity and seropositivity at altitudes under 2000 m, but not at 2000 m and above, primarily because bed net distribution programmes were focused specifically in lower altitudes where malaria transmission is known to be higher. Above 2000 m, IRS in the past 12 months was protective against RDT positivity, but was a significant risk factor for seropositivity. This may be due to the fact that certain IRS targeting programmes were implemented in areas ≥2000 m based on previous malaria outbreaks, and therefore may have masked the true effect of IRS spraying.

## Conclusions

As observed in other malarious areas [30–34], there was both RDT-positivity and seroprevalence heterogeneity across and within the different malaria transmission eco-epidemiological zones in Amhara, with overall higher rates for *P. falciparum* compared to *P. vivax* and substantially higher transmission in lower altitude areas. Progress made in Ethiopia during the last decade in decreasing malaria transmission should continue with effective interventions tailored to the socio-epidemiological characteristics of the area. Given the apparent importance of population mobility in the parasite transmission patterns, specific interventions targeting the source areas at lower elevations and the mobile population would be warranted to eliminate transmission in the highest altitude areas. Serology offers an opportunity to monitor transmission in low and seasonal transmission areas. Measuring changes in the transmission of malaria will be crucial to monitor progress toward malaria elimination, and serological surveys may be a critical tool in addition to infection detection to describe changes in malaria transmission.

## Additional files

**Additional file 1.** Age-seroconversion plots for antibody responses to *Plasmodium falciparum* antigens (left) and *P. vivax* antigens (right) for altitudes below 2000 metres (A), 2000 to 2500 metres (B), and greater than 2500 metres (C). Triangles represent deciles of observed data, solid lines represent the fit to the data of a reverse catalytic conversion model and broken blue lines provide the 95% confidence interval for this fit.

**Additional file 2.** Percent of RDT-positivity (*Plasmodium falciparum* and/or *P. vivax*), serology positivity and presence of fever by bed net ownership, bed net use and IRS use, in different altitude strata.

**Additional file 3.** Percent and odds ratios for RDT-positivity (*Plasmodium falciparum* and/or *P. vivax*) by sociodemographic characteristics and malaria risk factors, for altitudes <2000 metres and ≥2000 metres.

**Additional file 4.** Percent and odds ratios for seropositivity (*Plasmodium falciparum* and/or *P. vivax*) by sociodemographic characteristics and malaria risk factors, for altitudes <2000 metres and ≥2000 metres.

**Additional file 5.** Dot plots of seropositivity to *Plasmodium falciparum* antigens among children aged 1 to 5 years, by altitude.

## Abbreviations

AMA: apical membrane antigen
ANRSHB: Amhara National Regional State Health Bureau
DBS: dried blood spots
EA: enumeration area
IRS: indoor residual spraying
LLIN: long-lasting insecticide-treated nets
MSP: merozoite surface protein
TCC: The Carter Center
OD: optical density
OR: odds ratio
PDA: personal digital assistants
RDT: rapid diagnostic test
SCR: seroconversion rates
