# Supplementary material for: Current and cumulative malaria infections in a setting embarking on elimination: Amhara, Ethiopia
Source: Malar J. 2017 Jun 8;16:242. doi: 10.1186/s12936-017-1884-y (PMC5465535; doi:10.1186/s12936-017-1884-y)
Supplement: Supplementary file 1 — Additional file 1. Age-seroconversion plots for antibody responses to Plasmodium falciparum antigens (left) and P. vivax antigens (right) for altitudes below 2000 metres (A), 2000 to 2500 metres (B), and greater than 2500 metres (C). Triangles represent deciles of observed data, solid lines represent the fit to the data of a reverse catalytic conversion model and broken blue lines provide the 95% confidence interval for this fit. [file 12936_2017_1884_MOESM1_ESM.docx]

**Additional file 1**

**A**

**B**

**C**
