# Supplementary material for: Current and cumulative malaria infections in a setting embarking on elimination: Amhara, Ethiopia
Source: Malar J. 2017 Jun 8;16:242. doi: 10.1186/s12936-017-1884-y (PMC5465535; doi:10.1186/s12936-017-1884-y)
Supplement: Supplementary file 2 — Additional file 2. Percent of RDT-positivity (Plasmodium falciparum and/or P. vivax), serology positivity and presence of fever by bed net ownership, bed net use and IRS use, in different altitude strata. [file 12936_2017_1884_MOESM2_ESM.docx]

**Additional file 2**

|  | **<1000 meters** | | | **1000 to <2000 meters** | | | **2000 to <2200 meters** | | | **≥ 2200 meters** | | |
| --- | --- | --- | --- | --- | --- | --- | --- | --- | --- | --- | --- | --- |
|  | **% RDT+** | **% seropositive** | **%**  **with fever** | **%**  **RDT+** | **% seropositive** | **%**  **with fever** | **%**  **RDT+** | **% seropositive** | **%**  **with fever** | **%**  **RDT+** | **% seropositive** | **%**  **with fever** |
| **HH owns a bednet** |  |  |  |  |  |  |  |  |  |  |  |  |
| Yes | 6.4 | 52.8 | 18.0 | 1.5 | 33.2 | 10.2 | 1.3 | 33.4 | 14.0 | 0.3 | 17.7 | 7.2 |
| No | 21.5 | 62.1 | 23.3 | 3.3 | 37.1 | 11.9 | 0.9 | 24.7 | 13.5 | 0.6 | 19.6 | 8.0 |
| **Bednet use the previous night** |  |  |  |  |  |  |  |  |  |  |  |  |
| Yes | 6.6 | 54.8 | 19.4 | 1.1 | 31.8 | 11.3 | 1.6 | 32.4 | 14.7 | 0.3 | 19.1 | 7.4 |
| No | 9.6 | 53.9 | 18.5 | 2.6 | 35.7 | 10.8 | 1.1 | 29.5 | 13.5 | 0.4 | 18.1 | 7.5 |
| **HH received IRS in last 12 months** |  |  |  |  |  |  |  |  |  |  |  |  |
| Yes | 5.4 | 52.0 | 17.4 | 2.4 | 34.9 | 9.4 | 0.2 | 42.3 | 16.6 | 0.00 | 22.5 | 17.4 |
| No | 13.6 | 57.5 | 20.8 | 2.2 | 34.9 | 12.4 | 1.5 | 26.4 | 12.9 | 0.4 | 18.0 | 6.9 |
