# Supplementary material for: Current and cumulative malaria infections in a setting embarking on elimination: Amhara, Ethiopia
Source: Malar J. 2017 Jun 8;16:242. doi: 10.1186/s12936-017-1884-y (PMC5465535; doi:10.1186/s12936-017-1884-y)
Supplement: Supplementary file 3 — Additional file 3. Percent and odds ratios for RDT-positivity (Plasmodium falciparum and/or P. vivax) by sociodemographic characteristics and malaria risk factors, for altitudes <2000 metres and ≥2000 metres. [file 12936_2017_1884_MOESM3_ESM.docx]

**Additional file 3**

|  | **< 2000 meters** | | | | **≥ 2000 meters** | | | |
| --- | --- | --- | --- | --- | --- | --- | --- | --- |
|  | **RDT-negative**  **(N=4189)** | **RDT positive for *P. falciparum*, *P. vivax* or mixed**  **(N=149)** | | | **RDT-negative**  **(N=3512)** | **RDT positive for *P. falciparum*, *P. vivax* or mixed**  **(N=28)** | | |
|  | **n (%)** | **n (%)** | **Unadjusted OR (95% CI)†** | **Adjusted OR**  **(95% CI)‡** | **n (%)** | **n (%)** | **Unadjusted OR**  **(95% CI)†** | **Adjusted OR**  **(95% CI)‡** |
| **Sex** |  |  |  |  |  |  |  |  |
| Female | 2193 (52.4) | 65 (43.6) | Ref | Ref | 1832 (52.2) | 11 (32.3) | Ref | Ref |
| Male | 1996 (47.6) | 84 (56.4) | 1.42 (1.02 – 1.97) | 1.39 (0.99 – 1.95) | 1680 (47.8) | 17 (60.7) | 1.69 (0.79 – 3.61) | 1.62 (0.74 – 3.52) |
| **Age** |  |  |  |  |  |  |  |  |
| 6 months - 4 years | 1914 (56.7) | 81 (54.4) | Ref | Ref*** | 1300 (37.0) | 12 (42.9) | Ref | Ref |
| 5-9 years | 470 (11.2) | 26 (17.5) | 1.31 (0.83 – 2.06) | 1.50 (0.93 – 2.41) | 425 (12.1) | 3 (10.7) | 0.76 (0.21 – 2.72) | 1.01 (0.27 – 3.71) |
| 10-19 years | 511 (12.2) | 20 (13.4) | 0.92 (0.56 – 1.52) | 1.04 (0.62 – 1.74) | 559 (15.9) | 4 (14.3) | 0.78 (0.25 – 2.41) | 1.07 (0.33 – 3.49) |
| 20-39 years | 769 (18.4) | 15 (10.1) | 0.46 (0.26 – 0.80) | 0.44 (0.24 – 0.78) | 698 (19.9) | 4 (14.3) | 0.62 (0.20 – 1.93) | 0.68 (0.21 – 2.20) |
| ≥40 years | 525 (98.7) | 7 (1.3) | 0.32 (0.14 – 0.69) | 0.34 (0.15 – 0.75) | 530 (99.1) | 5 (0.9) | 1.02 (0.36 – 2.91) | 0.82 (0.27 – 2.45) |
| **Altitude** |  |  |  |  |  |  |  |  |
| <1000 meters | 1094 (26.1) | 70 (47.0) | Ref | Ref*** |  |  |  |  |
| 1000 to <2000 meters | 3095 (73.9) | 79 (53.0) | 0.25 (0.18 – 0.55) | 0.19 (0.13 – 0.27) |  |  |  |  |
| 2000 to <2200 meters |  |  |  |  | 1768 (50.3) | 21 (75.0) | Ref | Ref* |
| ≥2200 meters |  |  |  |  | 1744 (49.7) | 7 (25.0) | 0.34 (0.14 – 0.80) | 0.40 (0.16 – 0.98) |
| **Fever in the last 2 weeks** |  |  |  |  |  |  |  |  |
| No | 3702 (88.4) | 94 (63.1) | Ref | Ref | 3142 (89.5) | 13 (46.4) | Ref | Ref |
| Yes | 477 (11.4) | 55 (36.9) | 4.54 (3.21 – 6.42) | 3.97 (2.72 – 5.79) | 361 (10.3) | 15 (53.6) | 10.0 (4.74 – 21.27) | 7.62 (3.24 – 17.89) |
| **Malaria History§** |  |  |  |  |  |  |  |  |
| No | 4140 (98.3) | 142 (95.3) | Ref | Ref | 3479 (99.1) | 23 (82.1) | Ref | Ref* |
| Yes | 49 (1.2) | 7 (4.7) | 4.16 (1.85 – 9.36) | 0.81 (0.33 – 1.96) | 33 (0.9) | 5 (17.9) | 22.92 (8.21 – 63.94) | 3.78 (1.15 – 12.43) |
| **Bednet use the previous night** |  |  |  |  |  |  |  |  |
| No | 3325 (79.4) | 127 (85.2) | Ref | Ref | 2693 (76.7) | 20 (71.4) | Ref | Ref |
| Yes | 864 (20.6) | 22 (14.8) | 0.67 (0.42 – 1.05) | 0.85 (0.50 – 1.44) | 819 (23.3) | 8 (28.6) | 1.31 (0.58 – 3.00) | 1.30 (0.49 – 3.42) |
| **HH owns a bednet** |  |  |  |  |  |  |  |  |
| No | 1617 (38.6) | 77 (51.7) | Ref | Ref*** | 1187 (33.8) | 9 (32.1) | Ref | Ref |
| Yes | 2572 (61.4) | 72 (48.3) | 0.59 (0.42 – 0.82) | 0.39 (0.26 – 0.58) | 2325 (66.2) | 19 (67.9) | 1.08 (0.49 – 2.39) | 1.19 (0.48 – 2.97) |
| **HH received IRS in last 12 months** |  |  |  |  |  |  |  |  |
| No | 2012 (48.0) | 82 (55.0) | Ref | Ref | 2982 (84.9) | 27 (96.4) | Ref | Ref** |
| Yes | 2177 (52.0) | 67 (45.0) | 0.76 (0.54 – 1.05) | 0.79 (0.56 – 1.12) | 530 (15.1) | 1 (3.6) | 0.21 (0.03 – 1.54) | 0.12 (0.01 – 0.89) |

†Unadjusted odds ratio for the association between each risk factor and *P. vivax,* *P. falciparum*/mixed RDT result (versus a negative RDT result).

‡ Adjusted odds ratio for all the listed risk factors and a *P. vivax,* *P. falciparum*/mixed RDT result (versus a negative RDT result).

§ had a positive blood test for malaria or had taken antimalarial drug for the treatment of fever in the previous two weeks

Abbreviations: CI: confidence interval; HH: household; IRS: insecticide repellent spray; OR: odds ratio; Ref: Reference

*p<0.05, **p<0.01, ***p<0.001 from likelihood ratio test
