# Supplementary material for: Current and cumulative malaria infections in a setting embarking on elimination: Amhara, Ethiopia
Source: Malar J. 2017 Jun 8;16:242. doi: 10.1186/s12936-017-1884-y (PMC5465535; doi:10.1186/s12936-017-1884-y)
Supplement: Supplementary file 4 — Additional file 4. Percent and odds ratios for seropositivity (Plasmodium falciparum and/or P. vivax) by sociodemographic characteristics and malaria risk factors, for altitudes <2000 metres and ≥2000 metres. [file 12936_2017_1884_MOESM4_ESM.docx]

**Additional file 4**

|  | **< 2000 meters** | | | | **≥ 2000 meters** | | | |
| --- | --- | --- | --- | --- | --- | --- | --- | --- |
|  | **Seronegative**  **(N=2675)** | **Seropositive for *P. falciparum*, *P. vivax* or mixed**  **(N=1663)** | | | **Seronegative**  **(N=2679)** | **Seropositive for *P. falciparum*, *P. vivax* or mixed**  **(N=861)** | | |
|  | **n (%)** | **n (%)** | **Unadjusted OR (95% CI)†** | **Adjusted OR**  **(95% CI)‡** | **n (%)** | **n (%)** | **Unadjusted OR (95% CI)†** | **Adjusted OR**  **(95% CI)‡** |
| **Sex** |  |  |  |  |  |  |  |  |
| Female | 1409 (62.4) | 849 (37.6) | Ref | Ref*** | 1409 (76.5) | 434 (23.5) | Ref | Ref |
| Male | 1266 (60.9) | 814 (39.1) | 1.07 (0.94 – 1.21) | 1.29 (1.12 – 1.49) | 1270 (74.8) | 427 (25.2) | 1.09 (0.94 – 1.27) | 1.16 (0.99 – 1.37) |
| **Age** |  |  |  |  |  |  |  |  |
| 6 months - 4 years | 1626 (81.5) | 369 (18.5) | Ref | Ref*** | 1150 (87.7) | 162 (12.3) | Ref | Ref*** |
| 5-9 years | 359 (72.4) | 137 (27.6) | 1.68 (1.34 – 2.11) | 1.83 (1.45 – 2.31) | 354 (82.7) | 74 (17.3) | 1.48 (1.10 – 2.00) | 1.48 (1.09 – 2.01) |
| 10-19 years | 275 (51.8) | 256 (48.2) | 4.10 (3.34 – 5.03) | 4.70 (3.80 – 5.81) | 421 (74.8) | 142 (25.2) | 2.39 (1.86 – 3.08) | 2.48 (1.92 – 3.22) |
| 20-39 years | 247 (31.5) | 537 (68.5) | 9.58 (7.94 – 11.57) | 11.0 (9.02 – 13.40) | 428 (61.0) | 274 (39.0) | 4.54 (3.63 – 5.68) | 5.04 (4.00 – 6.34) |
| ≥40 years | 168 (31.6) | 364 (68.4) | 9.55 (1.70 – 11.84) | 11.34 (9.07 – 12.18) | 326 (60.9) | 209 (39.1) | 4.55 (3.58 – 5.78) | 4.87 (3.81 – 6.23) |
| **Altitude** |  |  |  |  |  |  |  |  |
| <1000 meters | 352 (45.8) | 416 (54.2) | Ref | Ref*** |  |  |  |  |
| 1000 to <2000 meters | 2323 (76.3) | 1247 (68.2) | 0.45 (0.38 – 0.53) | 0.34 (0.28 – 0.41) |  |  |  |  |
| 2000 to <2200 meters |  |  |  |  | 1248 (69.8) | 541 (30.2) | Ref | Ref*** |
| ≥2200 meters |  |  |  |  | 1431 (81.7) | 320 (18.3) | 0.52 (0.44 – 0.60) | 0.53 (0.45 – 0.63) |
| **Fever in the last 2 weeks** |  |  |  |  |  |  |  |  |
| No | 2399 (63.2) | 1397 (36.8) | Ref | Ref | 2418 (76.6) | 737 (23.4) | Ref | Ref |
| Yes | 271 (50.9) | 261 (49.4) | 1.65 (0.38 – 1.98) | 1.76 (1.41 – 2.19) | 253 (67.3) | 123 (32.7) | 1.60 (1.27 – 2.01) | 1.19 (0.91 – 1.55) |
| **Malaria History§** |  |  |  |  |  |  |  |  |
| No | 2649 (99.0) | 1633 (98.2) | Ref | Ref | 2663 (76.0) | 839 (24.0) | Ref | Ref** |
| Yes | 26 (1.0) | 30 (1.8) | 1.87 (1.10 – 3.18) | 1.06 (0.60 – 2.00) | 16 (42.1) | 22 (57.9) | 4.36 (2.28 – 8.35) | 3.09 (1.48 – 6.45) |
| **Bednet use the previous night** |  |  |  |  |  |  |  |  |
| No | 2123 (61.9) | 1329 (38.1) | Ref | Ref | 2070 (76.3) | 643 (23.7) | Ref | Ref |
| Yes | 552 (62.3) | 334 (37.7) | 0.97 (0.83 – 1.13) | 0.94 (0.77 – 1.14) | 609 (73.6) | 218 (26.4) | 1.15 (0.96 – 1.38) | 0.93 (0.75 – 1.14) |
| **HH owns a bednet** |  |  |  |  |  |  |  |  |
| No | 1037 (61.2) | 657 (38.8) | Ref | Ref** | 928 (77.6) | 268 (22.4) | Ref | Ref |
| Yes | 1638 (62.0) | 1006 (38.0) | 0.97 (0.86 – 1.10) | 0.80 (0.68 – 0.94) | 1751 (74.7) | 593 (25.3) | 1.17 (0.99 – 1.38) | 1.20 (0.99 – 1.45) |
| **HH received IRS in last 12 months** |  |  |  |  |  |  |  |  |
| No | 1293 (61.7) | 801 (38.3) | Ref | Ref | 2353 (78.2) | 656 (21.8) | Ref | Ref*** |
| Yes | 1382 (61.6) | 862 (58.4) | 1.01 (0.89 – 1.14) | 1.06 (0.92 – 1.23) | 326 (61.4) | 205 (38.6) | 2.26 (1.86 – 2.74) | 1.89 (1.52 – 2.35) |

†Unadjusted odds ratio for the association between each risk factor and *P. vivax,* *P. falciparum*/mixed RDT result (versus a negative RDT result).

‡ Adjusted odds ratio for all the listed risk factors and a *P. vivax,* *P. falciparum*/mixed RDT result (versus a negative RDT result).

§ had a positive blood test for malaria or had taken antimalarial drug for the treatment of fever in the previous 2 weeks

Abbreviations: CI: confidence interval; HH: household; IRS: insecticide repellent spray; OR: odds ratio; Ref: Reference

*p<0.05, **p<0.01, ***p<0.001 from likelihood ratio test
