# Supplementary material for: Current and cumulative malaria infections in a setting embarking on elimination: Amhara, Ethiopia
Source: Malar J. 2017 Jun 8;16:242. doi: 10.1186/s12936-017-1884-y (PMC5465535; doi:10.1186/s12936-017-1884-y)
Supplement: Supplementary file 5 — Additional file 5. Dot plots of seropositivity to Plasmodium falciparum antigens among children aged 1 to 5 years, by altitude. [file 12936_2017_1884_MOESM5_ESM.docx]

**Additional file 5**

**Legend:**

*P.falciparum* MSP

*P.falciparum* AMA
